# Early functional organization of the anterior and posterior hippocampus in the fetal brain

**DOI:** 10.1093/cercor/bhaf327

**Published:** 2025-12-09

**Authors:** Emily S Nichols, Sarah Al-Saoud, Michelle Fang, Roy Eagleson, Barbra de Vrijer, Charles McKenzie, Sandrine de Ribaupierre, Emma G Duerden

**Affiliations:** Faculty of Education, Western University, 1151 Richmond St, London, ON N6A 3K7, Canada; Western Institute for Neuroscience, Western University, 1151 Richmond St, London, ON N6A 3K7, Canada; Faculty of Education, Western University, 1151 Richmond St, London, ON N6A 3K7, Canada; Western Institute for Neuroscience, Western University, 1151 Richmond St, London, ON N6A 3K7, Canada; Physiology and Pharmacology, Schulich School of Medicine & Dentistry, Western University, 1151 Richmond St, London, ON N6A 3K7, Canada; Western Institute for Neuroscience, Western University, 1151 Richmond St, London, ON N6A 3K7, Canada; Electrical and Computer Engineering, Faculty of Engineering, Western University, 1151 Richmond St, London, ON N6A 3K7, Canada; Obstetrics & Gynaecology, Schulich School of Medicine & Dentistry, Western University, 1151 Richmond St, London, ON N6A 3K7, Canada; Division of Maternal, Fetal and Newborn Health, Children’s Health Research Institute, 800 Commissioners Rd East, London, ON N6C 2V5, Canada; Division of Maternal, Fetal and Newborn Health, Children’s Health Research Institute, 800 Commissioners Rd East, London, ON N6C 2V5, Canada; Medical Biophysics, Schulich School of Medicine & Dentistry, Western University, 1151 Richmond St, London, ON N6A 3K7, Canada; Western Institute for Neuroscience, Western University, 1151 Richmond St, London, ON N6A 3K7, Canada; Division of Maternal, Fetal and Newborn Health, Children’s Health Research Institute, 800 Commissioners Rd East, London, ON N6C 2V5, Canada; Clinical Neurological Sciences, Schulich School of Medicine & Dentistry, Western University, 1151 Richmond St, London, ON N6A 3K7, Canada; Faculty of Education, Western University, 1151 Richmond St, London, ON N6A 3K7, Canada; Western Institute for Neuroscience, Western University, 1151 Richmond St, London, ON N6A 3K7, Canada

**Keywords:** hippocampus, fetal brain, functional connectivity, functional magnetic resonance imaging, long-axis specialization

## Abstract

The hippocampus, in both children and adults, has shown functional specialization along its long axis, with the anterior region associated with emotional processing and the posterior region with spatial memory and navigation. This specialization is also reflected in separate patterns of functional connectivity, but it is unclear whether it is present before birth. Here, we collected resting-state fMRI data in 51 healthy third-trimester fetuses to examine long-axis functional specialization *in utero*. Using structural regions of interest in the anterior and posterior hippocampus, a seed-based connectivity analysis was performed. We identified distinct networks of functional organization for the anterior and posterior hippocampus. These patterns showed spatial organization and anticorrelation consistent with long-axis specialization. While less mature than those observed in postnatal human and preclinical models, the fetal patterns suggest that the foundation for hippocampal functional differentiation supporting early affective and cognitive processing is already present before birth.

**Key points**

- We used resting-state fMRI in the third trimester fetal brain to examine the functional projections of the anterior and posterior hippocampus.
- We identified distinct networks of functional organization that were independently related to the anterior and posterior hippocampus.
- The groundwork for the specificity of the hippocampus is being laid *in utero*, with functional anticorrelation contributing to the separation between long-axis segments.

## Introduction

The third trimester of human gestation represents a critical window for neural and cognitive development, including within the hippocampus which undergoes substantial development before birth (Grossman et al. 2006; Zhong et al. 2020). The hippocampus, a structure integral to memory, spatial navigation, emotion, and cognition, is organized along its long axis into the head, or anterior hippocampus (aHPC), and body and tail, or posterior hippocampus (pHPC), with each subdivision showing unique functional and structural organization and connectivity (Kier et al. 2004; Catenoix et al. 2011; Krebs et al. 2011; Poppenk and Moscovitch 2011). Understanding its functional projections at this early stage can provide unique insights into the foundation of cognitive and affective processes and neural network organization.

In adults, the anterior and pHPC are known to have distinct functional roles, with the anterior region associated with emotional processing and the posterior with spatial memory and navigation (Maguire et al. 2000; Grady 2020). This functional differentiation within the hippocampus is also reflected in its projections. Tracer studies performed in rodents and monkeys have shown that anatomically, direct connectivity between the anterior and posterior segments of the hippocampus is scarce. Instead, the two regions communicate indirectly through the entorhinal, perirhinal, and parahippocampal cortex, with aHPC showing stronger functional connectivity with the perirhinal cortex, while the pHPC shows stronger connections with the parahippocampal gyrus (Kahn et al. 2008a; Libby et al. 2012).

In addition to differences in their connections to the immediately surrounding medial temporal lobe structures, the aHPC and pHPC also project to separate cortical and subcortical regions. In humans, *ex vivo* and diffusion MRI studies have found direct connections between the aHPC and the amygdala and fusiform cortex, and indirect connections to the temporal pole, insula, gyrus rectus, and ventromedial prefrontal cortex through the uncinate fasciculus (Kier et al. 2004; Catenoix et al. 2011). Functionally, resting-state fMRI has revealed aHPC connections with the ventral tegmental area, nucleus accumbens, amygdala, hypothalamus, and anterolateral temporal lobes (Krebs et al. 2011). In contrast, the pHPC is functionally connected to the cuneus, precuneus, anterior and posterior cingulate cortex, inferior parietal cortex, and the thalamus (Poppenk and Moscovitch 2011; Poppenk et al. 2013). It is these distinctions between the aHPC and pHPC projections that are thought to underlie the functional specialization observed within the hippocampus itself (Poppenk et al. 2013). Additionally, the aHPC and pHPC contain different compositions of the hippocampal subfields, with a higher proportion of cornu ammonis (CA) fields 1–4 in the aHPC and a greater proportion of the dentate gyrus in the pHPC (Malykhin et al. 2010), and research in adults has shown differing patterns of connectivity along the long axis of the subfields (Dalton et al. 2019; Ezama et al. 2021).

Although this functional distinction in the hippocampus is less studied in children, a recent meta-analysis demonstrated that similar functional differentiation is present within the hippocampus in children as young as four years of age (Nichols et al. 2023a), and work in 4–10 years old has shown differing patterns of connectivity between regions, driven mostly by the aHPC (Blankenship et al. 2017). Hippocampal development is nonuniform across the longitudinal axis, with the aHPC maturing earlier than the pHPC, and this distinction is thought to underlie why aHPC-based learning such as statistical learning is possible early on, while episodic memory, dependent on the pHPC, tends to solidify later in childhood (Ghetti and Bunge 2012; Gómez and Edgin 2016). Despite these differences in rates of maturation, evidence suggests that both affective and semantic memory processes are present at birth. For example, at birth, neonates recognize the speech sounds of their mother’s language, indicating that memory formation for these sounds begins *in utero* (Byers-Heinlein et al. 2010). In a similar vein, neonates can distinguish between different affective prosodies (Cheng et al. 2012), show changes in heart rate variability to affective and nonaffective touch (Della Longa et al. 2021), and show signs of disgust to olfactory stimuli (Soussignan et al. 1997), suggesting again that the mechanisms supporting affective processing are in place at this time.

Advances in functional imaging (Fulford and Gowland 2009) and preprocessing pipelines (e.g., Nichols et al. 2023b; Wang et al. 2022) have made it possible to study fetal brain connectivity (Thomason et al. 2013; Correa et al. 2023), allowing the investigation of the earliest stages of human brain development. Using fetal fMRI allows us to determine when and how these functional specializations emerge and whether the groundwork for future cognitive abilities is in place before exposure to the external environment. By examining functional connectivity in the fetus, we can determine how the aHPC and pHPC interact with cortical and subcortical regions, and whether the neural groundwork for cognition, affect, and memory is being established *in utero*. This understanding could define how we view prenatal contributions to cognitive development, and the ontogeny of large-scale brain networks implicated in higher-order cognitive and affective functions.

In the present study, we used resting-state fMRI in the third trimester fetal brain to examine the functional projections of the anterior and pHPC. Using structural regions of interest in the aHPC and pHPC, a seed-based connectivity (SBC) analysis was performed, and functional correlations were sought in both cortical and subcortical regions. Given the cognitive and affective abilities present in neonates, it was expected that both the aHPC and pHPC would be functionally connected with their respective networks in the fetus during the third trimester; however, considering the different developmental trajectories, it was hypothesized that the aHPC would show greater connectivity than the pHPC within its network. Based on the seed-based functional connectivity analysis, we identified distinct networks of functional organization that were independently related to the aHPC and pHPC that closely resembled those found in both animal and human models, suggesting that the groundwork for hippocampus specialization is present before birth.

## Materials and methods

### Participants

Participants were 65 healthy women between the ages of 22 and 41 (*M* = 32.08, *SD* = 4.44) with singleton pregnancies in the third trimester of pregnancy (26.9 – 39.3 weeks, *M* = 32.62, *SD* = 3.25) recruited from the London, Canada, community. Inclusion criteria were a) 18 years of age or older, b) healthy singleton pregnancies, c) normal fetal growth, characterized by their healthcare provider as within the 10th to 90th percentile, and d) in the third trimester of gestation. Exclusion criteria were a) hypertension, b) gestational diabetes, c) contraindications to safely undergo noncontrast MRI, d) presence of a congenital anomaly, and e) concomitant substance use, including alcohol and cigarettes or other forms of tobacco. Participants were recruited through the community through advertisements. The Health Sciences Research Ethics Board at Western University approved the study (protocol #115440), and all individuals provided written informed consent prior to participating. The study procedures were carried out according to the Declaration of Helsinki.

### MRI acquisition and preprocessing

Imaging was conducted with the participant in a left lateral position on a 3T (General Electric [GE], Milwaukee, WI; MR7500) MRI with a 32-channel torso coil at the Translational Imaging Research Facility at Robarts Research Institute, Western University, London, Canada. T2*-weighted resting-state fMRI scans were acquired axial to the fetal brain using an echo planar imaging (EPI) sequence (TR = 2000 ms, TE = 60 ms, flip angle = 90°, voxel size = 3.75*3.75*4 mm^3^, FOV = 240 mm) with 24 slices per volume, providing full coverage of the fetal cerebrum and cerebellum. A total of 110 volumes were acquired for each participant. To ensure the stability of the MR signal, two functional volumes were run but then discarded prior to the start of image acquisition.

Anatomical images were acquired using a T2-weighted sequence that consisted of 2D low-resolution stacks of images. Participants were imaged to acquire at least 3 single shot fast spin echo (SSFSE) images (TR > 1,000 ms, TE = 80 ms, FOV = 38–44 mm, 0.74 × 0.74 (coronal and sagittal) or 0.86 × 0.86 (axial) matrix, slice thickness = 5 mm, 19–25 slices), with one in each *x*, *y*, and *z* plane relative to the fetal brain. Images were then constructed into 3D volumes resampled to 1 mm isotropic resolution using NiftyMIC (Ebner et al. 2020), correcting for motion and bias field inhomogeneities. Anatomical data were then segmented into grey matter, white matter, and CSF tissue classes using SPM unified segmentation and normalization algorithm (Ashburner and Friston 2005; Ashburner 2007) with the default IXI-549 tissue probability map template.

Functional scans were first masked using *funcmasker-flex* (Nichols, Correa et al. 2023) to isolate the fetal brain from surrounding tissue. Data were then motion-corrected using Advanced Normalization Tools (Avants et al. 2011). FEAT (FMRI Expert Analysis Tool) Version 6.00, part of FSL (FMRIB's Software Library, www.fmrib.ox.ac.uk/fsl) was then used to apply a second round of motion correction using MCFLIRT (Jenkinson et al. 2002); slice-timing correction using Fourier-space time-series phase-shifting; spatial smoothing using a Gaussian kernel of FWHM 5 mm; grand-mean intensity normalisation of the entire 4D dataset by a single multiplicative factor; and high pass temporal filtering (Gaussian-weighted least-squares straight line fitting, with sigma = 50.0 s). Registration to the high-resolution structural images was carried out using FLIRT (Jenkinson and Smith 2001; Jenkinson et al. 2002) using a full linear search and 12 degrees of freedom. Registration from high-resolution structural to a standard space 36-week fetal template (Gholipour et al. 2017) was then further refined using FNIRT nonlinear registration with a full search and 12 degrees of freedom (Andersson et al. 2007a, 2007b). A 36 week template was used to align with previous work in fetuses (Karolis et al. 2025). Functional scans that had a maximum displacement greater than 3 mm of motion in any plane and in pitch, roll, and yaw were discarded (*n =* 14), resulting in a final dataset of *n =* 51. The average level of motion was not correlated with the gestational age of the fetus (*r*(49) = -0.20, *P* = 0.155). The maximum level of motion in the final dataset of 51 fetuses was 2.98 mm, and the mean level of motion was 0.49 mm (*SD* = 0.20).

Functional data were denoised using a standard denoising pipeline (Nieto-Castanon 2020b), including the regression of potential confounding effects characterized by white matter time series (five CompCor noise components), CSF time series (five CompCor noise components), session effects and their first order derivatives (two factors), and linear trends (two factors) within each functional run, followed by bandpass frequency filtering of the BOLD time series (Hallquist et al. 2013) between 0.008 Hz and 0.09 Hz. CompCor (Behzadi et al. 2007; Chai et al. 2012) noise components within white matter and CSF were estimated by computing the average BOLD signal as well as the largest principal components orthogonal to the BOLD average within each subject's eroded segmentation masks. From the number of noise terms included in this denoising strategy, the effective degrees of freedom of the BOLD signal after denoising were estimated to average 31.8 across all participants (Nieto-Castanon 2022).

### Anterior and posterior hippocampus segmentation

**Fig. 1:**
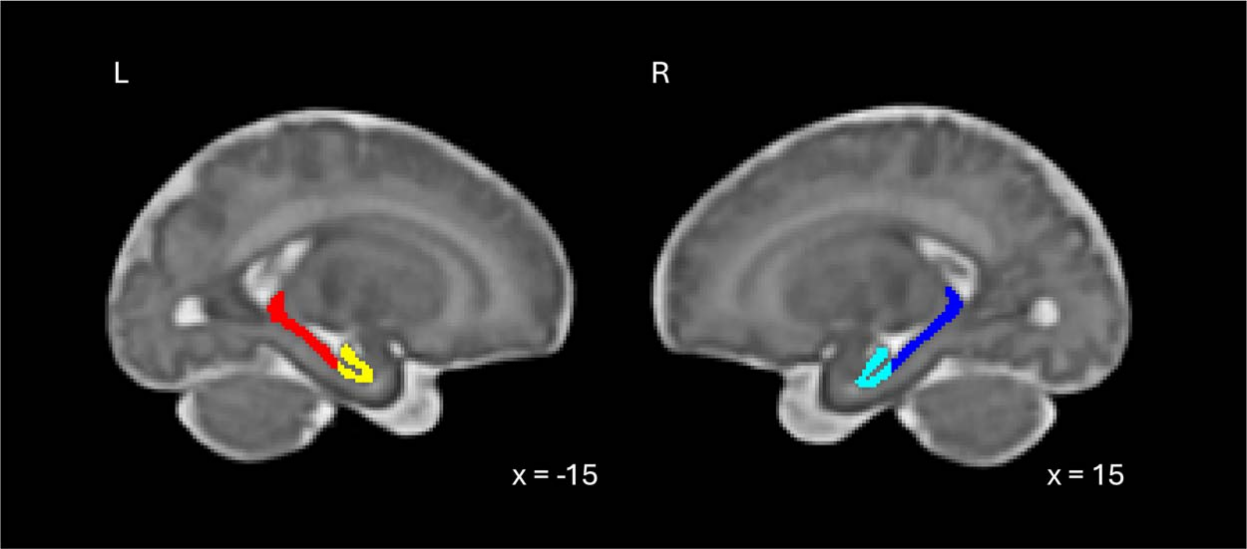
Segmentation of the anterior and posterior divisions of the left (yellow/red) and right (light/dark blue) hippocampus in fetal template space at 36 weeks gestational age.

The anterior and posterior regions of the hippocampus were segmented on the hippocampal region from a fetal brain structural atlas (Gholipour et al. 2017)using the protocol most used in hippocampal head/tail segmentation (N. V. Malykhin et al. 2007; Poppenk et al. 2013; Yushkevich et al. 2010) and are shown in Figure 1. The border between the aHPC and pHPC was defined by the presence or absence of the uncus. Specifically, the last slice in which the uncus is visible was defined as the last slice of the aHPC. On the fetal template, at or anterior to *y*=4 was considered aHPC (Poppenk et al. 2013). The left and right aHPC and pHPC segmentations were then used as seed regions for the functional connectivity analysis.

### Seed-based functional connectivity analysis

Functional connectivity analyses of fMRI data were performed using CONN (Whitfield-Gabrieli and Nieto-Castanon 2012) (RRID:SCR_009550) release 22.v2407 (Nieto-Castanon and Whitfield-Gabrieli 2022) and SPM (Penny et al. 2011) (RRID:SCR_007037) release 25.25.01.02. SBC maps were estimated by characterizing the spatial pattern of functional connectivity with a seed area. Seed regions included the left aHPC, right aHPC, left pHPC, and right pHPC, as defined using the fetal brain atlas (Gholipour et al. 2017). Functional connectivity strength was represented by Fisher-transformed bivariate correlation coefficients from a weighted general linear model (weighted-GLM; Nieto-Castanon 2020c), estimated separately for each seed area and target voxel, modeling the association between their BOLD signal time series. In order to compensate for possible transient magnetization effects at the beginning of each run, individual scans were weighted by a step function convolved with an SPM canonical hemodynamic response function and rectified. Motion was included as a covariate in the first-level analysis.

Group-level analyses were performed using a General Linear Model (GLM; Nieto-Castanon, 2020e). For each individual voxel a separate GLM was estimated, with first-level connectivity measures at this voxel as dependent variables (one independent sample per subject and one measurement per task or experimental condition, if applicable), and *z*-scored gestational age and fetus sex as independent variables. Voxel-level hypotheses were evaluated using multivariate parametric statistics with random-effects across subjects and sample covariance estimation across multiple measurements. Inferences were performed at the level of individual clusters (groups of contiguous voxels). Cluster-level inferences were based on parametric statistics from Gaussian Random Field theory (Worsley et al. 1996; Nieto-Castanon 2020a). Results were thresholded using a combination of a cluster-forming *P* < 0.001 voxel-level threshold, and a familywise corrected p-FDR < 0.05 cluster-size threshold (Chumbley et al. 2010).

## Results

### Demographic information

After exclusions, the final sample consisted of 51 pregnant individuals aged 22–41 (*M* = 31.78, *SD* = 4.77). There were 30 female and 21 male fetuses, ranging in gestational age from 27.7 to 39.3 weeks. Average years of maternal education was 16.63 (*SD* = 2.45), or equivalent to a Bachelor’s degree, and ranged from completion of Canadian high school (12 years) to a Doctoral degree (22 years). Demographic information for both the full and final samples is summarized in Table 1.

**Table 1:** Demographic information.

	Full sample, n or *M* (*SD*)	Final sample, n, or *M* (*SD*)
n	65	51
Age (years)	32.08 (4.44)	31.78 (4.77)
Education (years)	16.58 (2.57)	16.63 (2.45)
Gravida	1.95 (0.99)	2.04 (1.04)
Gestational age of fetus (weeks)	32.62 (3.25)	33.14 (3.03)
Sex of fetus (# female)	31	30

### Seed-based functional connectivity analysis

**Fig. 2:**
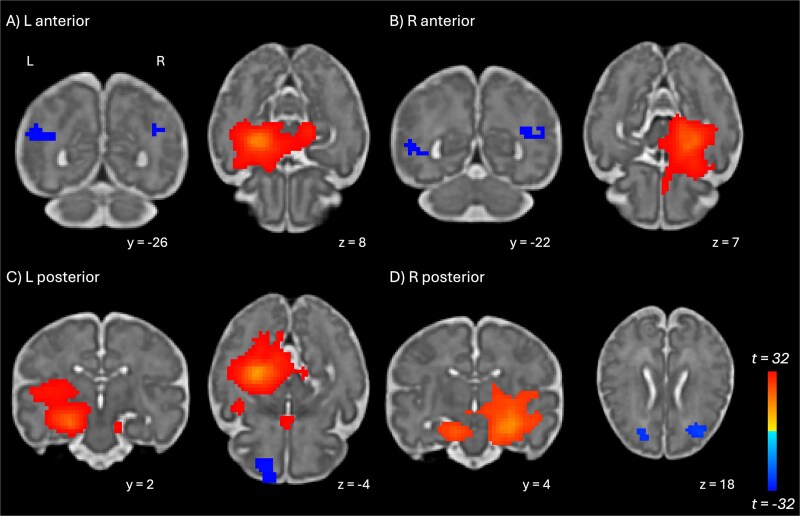
Functional connectivity of the hippocampus with seed regions in the a) left aHPC; b) right aHPC; c) left pHPC; and d) right HPC. L=left, coordinates in fetal template space.

Distinct connectivity patterns for all four regions of interest (left, right a/pHPC) were identified based on SBC analyses (Table 2). Examining anterior regions, both the left and right aHPC showed the most extensive connectivity with regions immediately adjacent to the seed. Although the peak was within the aHPC itself, the cluster included the ipsilateral amygdala and parahippocampal gyrus. Both seed regions in the aHPC showed negative correlations with activity in the territory of the bilateral angular gyrus (Figure 2). Although peak correlation values were found within temporal, parietal, and occipital lobes, the location of the correlated activity was very close between the two seed regions in aHPC. Finally, both aHPC seeds also showed negative correlations with activity in the right frontal lobe. Full multislice connectivity patterns for each seed region are shown in Figs. S1–S4.

**Table 2:** Clusters of significant connectivity for each hippocampus seed region.

Seed	Region	x	y	z	Size (mm^3^)	*t*(48)	*P*
Left aHPC	Left aHPC	−16	4	−12	2174	31.38	< 0.001
	Right middle frontal lobe	22	42	6	87	−5.32	< 0.001
	Left middle temporal lobe	−26	−22	10	63	−5.35	0.001
	Right angular gyrus	26	−32	18	33	−4.77	0.021
Right aHPC	Right aHPC	14	8	−12	2062	32.09	< 0.001
	Right superior frontal lobe	16	20	32	69	−4.33	0.002
	Left inferior occipital lobe	−28	−30	0	53	−4.46	0.005
	Right middle temporal lobe	30	−20	8	31	−4.07	0.038
Left pHPC	Left pHPC	−18	−6	−4	2337	21.15	< 0.001
	Left middle orbitofrontal lobe	−12	46	−6	130	−5.52	< 0.001
	Left olfactory lobe	−2	24	−8	55	6.60	0.005
Right pHPC	Right pHPC	16	−4	−2	2793	19.69	< 0.001
	Left inferior occipital lobe	−28	−26	0	212	−5.14	< 0.001
	Right middle frontal lobe	22	38	18	187	−5.58	< 0.001
	Left superior orbitofrontal lobe	−8	54	−2	80	−4.56	< 0.001
	Right calcarine sulcus	10	−46	0	74	−4.94	< 0.001
	Left middle temporal lobe	−40	−16	−2	48	−4.45	0.005
	Left middle frontal lobe	−12	40	10	47	−4.33	0.005

*Note*: coordinates are in fetal template space and denote area of peak connectivity within a cluster; *P*-values corrected using a false discovery rate.

Examining the posterior regions, both the left and right pHPC again showed peak connectivity with regions immediately adjacent to the seeds, including the parahippocampal gyrus, precuneus, and thalamus, and extending into the territory of the insula. Negative correlations were observed with cortical regions in the frontal, temporal, and occipital lobes, but not in the parietal lobe. The left pHPC also showed a positive correlation with the ipsilateral olfactory lobe.

When examining the effects of the control variables, namely sex and gestational age, neither one showed a significant relationship with aHPC or pHPC connectivity.

### Sensitivity analysis

In order to further explore the lack of age effects, a sensitivity analysis was conducted in fetuses above the median gestational age of the sample (*n =* 26). Given the analysis in older fetuses included only 26 participants, it was underpowered, thus the results must be interpreted with caution. The cluster-forming voxel-level threshold was changed to *P* < 0.005 (from .001) to account for the smaller sample size but the familywise corrected p-FDR cluster-size threshold was kept at *P <* 0.05. The results remained largely unchanged, and are shown in Figs. S5–S9. Peak cluster summary statistics, including the direction of the effects, are also shown in Table S1.

## Discussion

In this study, we sought to understand hippocampal specificity early in life by examining connectivity patterns of the anterior and pHPC in the third-trimester fetus. By using a seed-based approach, we determined which brain areas were functionally connected with the hippocampus, divided along its long axis. We found distinct patterns of connectivity for each seed region, with the overarching results reflecting connectivity with cortical, medial temporal, and midbrain regions. In addition to separate networks of connectivity, the results also revealed overlapping areas within hemisphere and within anterior/posterior division, particularly in the midbrain. Overall, the results were consistent with our hypotheses, with evidence suggesting the emergence of early, less mature long-axis connections in the third trimester. One key finding emerged, in that in addition to positive correlations with the expected regions for aHPC and pHPC separately, both showed anticorrelations with areas generally connected to the opposing hippocampal segment.

The left and right aHPC showed overlapping and mirrored connectivity patterns. Although the coordinates of peak connectivity were in the respective seed regions, the region of interest extended to include the ipsilateral perirhinal cortex, amygdala, olfactory lobe, inferior temporal lobe, fusiform gyrus, and midbrain. Connectivity with the left aHPC also extended into the contralateral hemisphere, through the midbrain and into the right aHPC and medial temporal lobe. This pattern is consistent with what has been shown both structurally (Kier et al. 2004; Catenoix et al. 2011) and functionally (Krebs et al. 2011), indicating that the mechanisms supporting processes associated with the aHPC, such as statistical learning and early emotional processing (Murty et al. 2010; Karim and Perlman 2017; Ellis et al. 2021), are present *in utero*.

In addition to positive correlations with medial temporal and inferior frontal regions, we also observed negative correlations between the aHPC and angular, middle temporal, middle frontal, and superior frontal regions. The angular gyrus and middle temporal gyrus underlie spatial processing, memory retrieval, and semantic memory more generally (Howard et al. 2005; Davey et al. 2016; Bellana et al. 2023; Seghier 2023). The middle frontal lobe is involved in inhibitory control, especially over memory-related areas like the hippocampus (Munakata et al. 2011), while the posterior superior frontal lobe is associated with executive function (Li et al. 2013). Notably, these are all regions that are typically connected, both structurally and functionally, with the pHPC (Kahn et al. 2008b; Poppenk and Moscovitch 2011). Anti-correlation is often characterized as a suppression of activity or deactivation within one region when another region is active (Fox et al. 2005). In the context of long-term potentiation and depression (see Clapp et al. 2012, for a review of animal and human literature), then this anticorrelation might lead to the pruning or suppression of connections between these regions. It is possible, then, that the groundwork for the specificity of the aHPC and pHPC is being laid *in utero*, with functional anticorrelation contributing to this separation between hippocampal long-axis segments. Alternatively, the anticorrelation observed in the angular and middle temporal regions could be related to early default mode network formation. The default mode network has been previously observed in fetuses (Correa et al. 2023) and is known to show negative correlations with other brain networks in adults (Greicius et al. 2003). However, the frontal regions observed here are more lateral than those that comprise the default mode network, and previous work has shown positive, rather than negative, correlations with other networks in the fetus (Scheinost et al. 2024), making it unlikely that this explains their observed anticorrelation. Thus, it remains possible that the anticorrelation observed here is related to the early development of functional specificity in the hippocampus.

A complementary connectivity pattern emerged when examining the pHPC. In line with research in older populations, we observed positive functional connectivity between the pHPC and the parahippocampal gyrus, insula, thalamus, temporal pole, Heschl’s gyrus, and medial regions of the middle temporal gyrus (Kahn et al. 2008b; Libby et al. 2012; Fritch et al. 2021). This same region of interest also extended into ventral and middle occipital areas, including the lingual gyrus, precuneus, cuneus, and calcarine sulcus. This network of regions again lines up well with previous research in pHPC connectivity (Poppenk and Moscovitch 2011; Poppenk et al. 2013). Notably however, these correlations did not extend into parietal regions, an area whose connections with the hippocampus are typically associated with spatial processing and navigation (Whitlock et al. 2008; Rolls et al. 2022), as well as episodic memory (Sestieri et al. 2017). The pHPC is known to mature later than the aHPC (Lavenex and Banta Lavenex 2013); thus, it is not surprising to find patterns of connectivity in the fetus that appear less mature than in older children or adults. The functional correlation with temporal and occipital but not parietal regions may contribute to the phenomenon of infantile amnesia (Rubin 2000), in which episodic memories are not retained early in life, while other types of memory, such as semantic, progress steadily during this period (Morris et al. 2010; Bauer and Larkina 2014; Bauer 2015).

Similar to the aHPC, we observed a network of regions showing negative correlations with the pHPC. These included orbitofrontal, middle frontal, calcarine, and inferior temporal and occipital regions. Interestingly, both the left and right pHPC showed anticorrelation with the left orbitofrontal cortex. The orbitofrontal cortex has been heavily studied in relation to affect, especially in emotion, motivation, and reward processing (Rempel-Clower 2007; Rolls et al. 2020), and direct structural connections exist between it and the amygdala (Sharpe and Schoenbaum 2016; Rolls et al. 2023). The observed anticorrelation may again reflect suppression of the connections between this area and the pHPC, laying the groundwork for disparate specialization between hippocampal long-axis segments.

Importantly, the hippocampus is an experience-sensitive structure, and work in children, preterm-born infants, and rodent models have demonstrated that it is particularly vulnerable to early stress (Poeggel et al. 2003; Dahmen et al. 2017; Volpe 2019; Reh et al. 2020). In terms of functional connectivity, early-life stress has been linked to lower coupling between the hippocampus and surrounding regions including the parahippocampual and fusiform gyri (Lammertink et al. 2022), and both weaker hippocampal-cingulate connectivity and lower infant memory outcomes (Scheinost et al. 2020). The present results demonstrate that differential long-axis connectivity is present during the third trimester *in utero*, a period of rapid brain development, indicating that prenatal exposures to stress such as maternal anxiety and depression could influence long-axis specialization during this period. Future research should seek to investigate the role of such early exposures on the hippocampus, considering its sensitivity.

Although the presence of functional connectivity networks has been well-established in the fetal brain (van den Heuvel and Thomason 2016; van den Heuvel et al. 2018; Thomason 2020), the third trimester covers a three-month period in which large developmental changes occur (Clouchoux et al. 2012; Bouyssi-Kobar et al. 2016; Nichols et al. 2024). While we did not observe any effect of age in our functional connectivity model, we cannot rule out that a more cohesive sample in terms of age, would produce different results. Despite the relatively large age range, the correlations we observed were robust and fit well with research in older populations. Additionally, while examining connectivity patterns between the aHPC and pHPC is informative to models of hippocampal development *in utero*, the resolution and quality of fetal anatomical and fMRI data limits how fine-grained the analysis can be. Analysis of individual hippocampal subfield connectivity would further inform our understanding of development, and will be possible with advances in fetal imaging. Finally, a large number of fetal scans had to be excluded due to motion, potentially limiting the generalizability of our findings, however it is unclear whether the differences in level of motion would be meaningful in terms of hippocampal connectivity.

## Conclusion

Investigating anterior and posterior hippocampal connectivity in the third trimester fetus offers a unique opportunity to address fundamental questions about brain development. Given the importance of the hippocampus in human cognitive and affective function, mapping its early functional development can help us understand both the typical and atypical brain. Here, we showed that the groundwork for hippocampus specialization is present *in utero*, with networks reflecting similar but immature connectivity to that present later in childhood and adulthood.

## Supplementary Material

supplemental_materials_bhaf327

## Data Availability

The data that support the findings of this study are available on request from the corresponding author. The data are not publicly available due to privacy or ethical restrictions.
