# Supplementary material for: Early functional organization of the anterior and posterior hippocampus in the fetal brain
Source: Cereb Cortex. 2025 Dec 9;35(12):bhaf327. doi: 10.1093/cercor/bhaf327 (PMC12687868; doi:10.1093/cercor/bhaf327)
Supplement: supplemental_materials_bhaf327 [file supplemental_materials_bhaf327.docx]

**Supplementary materials**

**Early Functional Organization of the Anterior and Posterior Hippocampus in the Fetal Brain**

Emily S. Nichols*^1,2^, Sarah Al-Saoud^1,2^, Michelle Fang^3^, Roy Eagleson^2,4^, Barbra de Vrijer^5,6^, Charles McKenzie^6,7^, Sandrine de Ribaupierre^2,6,8^, Emma G. Duerden^1,2^

Affiliations:

^1^Faculty of Education, Western University, London, Canada

^2^Western Institute for Neuroscience, Western University, London, Canada

^3^Physiology and Pharmacology, Schulich School of Medicine & Dentistry, Western University, London, Ontario, Canada

^4^Electrical and Computer Engineering, Faculty of Engineering, Western University, London, Canada

^5^Obstetrics & Gynaecology, Schulich School of Medicine & Dentistry, Western University, London, Ontario, Canada

^6^Division of Maternal, Fetal and Newborn Health, Children’s Health Research Institute

^7^Medical Biophysics, Schulich School of Medicine & Dentistry, Western University, London, Ontario, Canada

^8^Clinical Neurological Sciences, Schulich School of Medicine & Dentistry, Western University, London, Ontario, Canada

*Corresponding author:

Emily S. Nichols, PhD

enicho4@uwo.ca

Applied Psychology, Faculty of Education

Room 1131, 1137 Western Rd
London, Ontario N6G 1G7

ORCID: 0000-0003-0541-9233

Figure S1. Functional connectivity of the hippocampus with seed region in the left aHPC, displayed in neurological space (left=left). Z-coordinates shown in fetal template space.

Figure S2. Functional connectivity of the hippocampus with seed region in the right aHPC, displayed in neurological space (left=left). Z-coordinates shown in fetal template space.

Figure S3. Functional connectivity of the hippocampus with seed region in the left pHPC, displayed in neurological space (left=left). Z-coordinates shown in fetal template space.

Figure S4. Functional connectivity of the hippocampus with seed region in the right pHPC, displayed in neurological space (left=left). Z-coordinates shown in fetal template space.

Sensitivity analysis results


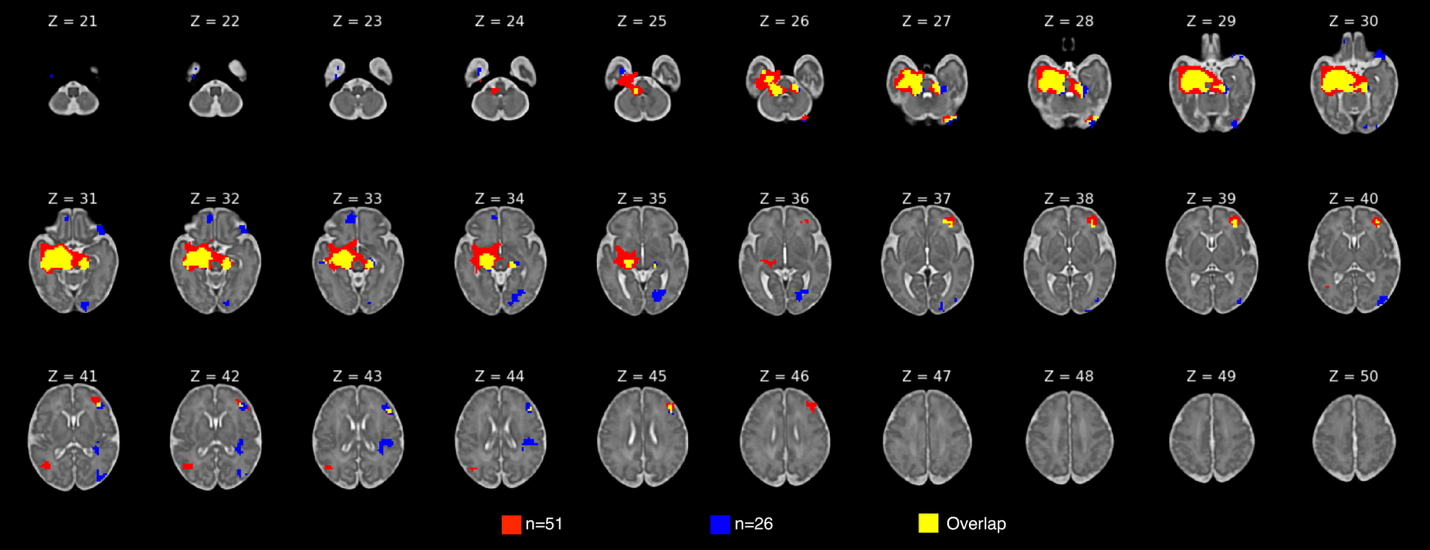
 Figure S5. Sensitivity analysis results. Conjunction maps of the functional connectivity of the hippocampus with seed region in the left aHPC, displayed in neurological space (left=left). Areas in red show significant correlations and anticorrelations with the full sample (n=51), and areas in blue show significant correlations and anticorrelations with only the older half of the sample (n=26). Areas in yellow show the overlap in significant results between the two analyses. Z-coordinates shown in fetal template space.

Figure S6. Sensitivity analysis results. Conjunction maps of the functional connectivity of the hippocampus with seed region in the right aHPC, displayed in neurological space (left=left). Areas in red show significant correlations and anticorrelations with the full sample (n=51), and areas in blue show significant correlations and anticorrelations with only the older half of the sample (n=26). Areas in yellow show the overlap in significant results between the two analyses. Z-coordinates shown in fetal template space.

Figure S7. Sensitivity analysis results. Conjunction maps of the functional connectivity of the hippocampus with seed region in the left pHPC, displayed in neurological space (left=left). Areas in red show significant correlations and anticorrelations with the full sample (n=51), and areas in blue show significant correlations and anticorrelations with only the older half of the sample (n=26). Areas in yellow show the overlap in significant results between the two analyses. Z-coordinates shown in fetal template space.

Figure S8. Sensitivity analysis results. Conjunction maps of the functional connectivity of the hippocampus with seed region in the right pHPC, displayed in neurological space (left=left). Areas in red show significant correlations and anticorrelations with the full sample (n=51), and areas in blue show significant correlations and anticorrelations with only the older half of the sample (n=26). Areas in yellow show the overlap in significant results between the two analyses. Z-coordinates shown in fetal template space.

| Table S1. Clusters of significant connectivity for each hippocampus seed region in older fetuses (n=26) | | | | | | | |
| --- | --- | --- | --- | --- | --- | --- | --- |
| Seed | Region | x | y | z | Size (mm^3^) | *t*(23) | *p* |
| Left aHPC | Left aHPC | -16 | 4 | -12 | 1139 | 23.66 | < .001 |
|  | Right lingual gyrus | 14 | -30 | -2 | 111 | -4.59 | .002 |
|  | Right pars triangularis | 26 | 34 | 12 | 88 | -4.33 | .005 |
|  | Right rolandic operculum | 24 | 0 | 14 | 81 | 4.72 | .006 |
|  | Right inferior orbitofrontal lobe | 28 | 32 | -8 | 51 | -3.95 | .041 |
|  | Right middle occipital lobe | 28 | -36 | 8 | 48 | -4.32 | .041 |
|  | Left gyrus rectus | -6 | 46 | -8 | 46 | -5.93 | .041 |
|  | Right inferior occipital lobe | 20 | -34 | -18 | 45 | -4.54 | .041 |
| Right aHPC | Right aHPC | 14 | 8 | -12 | 1964 | 26.95 | < .001 |
|  | Right inferior occipital lobe | 22 | -32 | -4 | 341 | -5.58 | < .001 |
|  | Left inferior occipital lobe | -24 | -42 | -8 | 144 | -5.33 | < .001 |
| Left pHPC | Left pHPC | -20 | -4 | -4 | 1389 | 15.79 | .005 |
|  | Left superior orbitofrontal lobe | -8 | 54 | 2 | 80 | -5.26 | < .001 |
|  | Right pHPC | 8 | -6 | -2 | 61 | 5.75 | < .001 |
|  | Right insula | 18 | 30 | 6 | 35 | -5.71 | .009 |
| Right pHPC | Right pHPC | 16 | -12 | 0 | 2656 | 13.98 | < .001 |
|  | Right middle frontal lobe | 24 | 38 | 18 | 311 | -6.84 | < .001 |
|  | Right inferior occipital lobe | 12 | -46 | -4 | 113 | -5.70 | .001 |
|  | Left superior orbitofrontal lobe | -8 | 54 | -2 | 108 | -5.73 | .001 |
|  | Left lingual gyrus | -12 | -26 | -10 | 65 | -4.34 | .021 |
|  | | | | | | | |
